# A systematic review of the association between insulin resistance surrogate indices and bone mineral density

**DOI:** 10.3389/fendo.2024.1499479

**Published:** 2024-12-18

**Authors:** Amirhossein Shirinezhad, Alireza Azarboo, Amirhossein Ghaseminejad-Raeini, Fatemeh Kanaani Nejad, Negar Zareshahi, Sheyda Mohtasham Amiri, Yasamin Tahmasebi, Amir Human Hoveidaei

**Affiliations:** ^1^ School of Medicine, Tehran University of Medical Sciences, Tehran, Iran; ^2^ Medical Imaging Research Center, Shiraz University of Medical Sciences, Shiraz, Iran; ^3^ Department of Medicine, Islamic Azad University Tehran Medical Sciences, Tehran, Iran; ^4^ Sports Medicine Research Center, Neuroscience Institute, Tehran University of Medical Sciences, Tehran, Iran

**Keywords:** insulin resistance, bone mineral density, HOMA-IR, TyG index, VAI, systematic review, osteoporosis, BMD

## Abstract

**Background:**

The relationship of insulin resistance with bone mineral density (BMD) remains unclear, offering an opportunity for novel indices to shed light on the matter. The aim of this review was to evaluate the association between surrogate indices of insulin resistance and BMD.

**Methods:**

A systematic review was conducted to evaluate observational studies that examined the relationship between insulin resistance surrogate indices and BMD in adults. Databases including PubMed, Web of Science, Scopus, and Embase were searched. Quality assessment was performed using Joanna Briggs Institute (JBI) critical appraisal tools.

**Results:**

This systematic review included 27 cohorts and cross-sectional studies with 71,525 participants to assess the potential link between insulin resistance surrogate indices like HOMA-IR, HOMA-β, TyG, TyG-BMI, TyG-WtHR, and TyG-WC, along with METS-IR, and VAI, and BMD at various sites. There seems to be no link between BMD and the HOMA index, despite being extensively studied in various studies (adjusted β ranging from -0.49 to 0.103). Most literature suggests that a higher TyG index is associated with decreased BMD levels (adjusted β ranging from -0.085 to 0.0124). Despite limited evidence, other insulin resistance indices such as VAI (adjusted β ranging from 0.007 to 0.016), TyG-BMI (adjusted β ranging from 0.002 to 0.415), METS-IR (adjusted β ranging from 0.005 to 0.060), TyG-WtHR (β = 0.012) and TyG-WC (β = 0.0001) have shown a positive association with BMD in a few studies.

**Conclusion:**

This systematic review emphasizes the intricate connection between insulin resistance and BMD. The lack of ability to perform a meta-analysis and the dependence on cross-sectional studies hinder the robustness of the findings, hence necessitating well-designed longitudinal studies.

**Systematic review registration:**

https://www.crd.york.ac.uk/prospero/, identifier CRD42024512770.

## Introduction

Osteoporotic fractures are associated with low BMD, making them a risk factor. The age-standard rate of osteoporosis incidence in 2019 was 49.2 million (1). The amount of global deaths and DALYs linked to low bone mineral density rose by 111.16% and 93.82% from 207,367 and 8,588,936 in 1990 to 437,884 and 16,647,466 in 2019 (2). Several variables can influence bone health and contribute to the development of osteoporosis (3). While certain characteristics like age, sex, or ethnicity have an inescapable impact on bone health, various modifiable factors, such as weight, alcohol consumption, or a sedentary lifestyle, can be improved by appropriate diet and physical activity to enhance bone condition (4). Multiple investigations have assessed the substantial impact of insulin resistance on various health-related conditions, including bone health.

The literature was clued into the inverse association of insulin resistance and bone health when the hypothesis of the negative impact of insulin resistance on bone remodeling was first presented (5, 6). Visceral fat mass (7), an altered lipid profile (8), and metabolic syndrome (9) are additional factors that might negatively impact bone health, leading to osteoporosis and an increased risk of fractures in the population. These parameters are sometimes referred to as surrogate indices for insulin resistance (10, 11). Multiple investigations have assessed the substantial impact of insulin resistance surrogate indices on various health-related conditions, such as vascular damage (12), hypertension (13), and metabolic syndrome (14). Furthermore, the correlation between these factors and bone health is receiving significant attention and has been thoroughly examined in several research studies (15, 16).

Given that the findings of studies on the association of insulin resistance and its surrogates with BMD are not consistent (17–20) and lack systematic reviews on this association to summarize the available evidence, the objective of this study was to assess the impact of surrogate indices of insulin resistance, including Homeostatic Model Assessment for Insulin Resistance (HOMA-IR), Homeostatic Model Assessment for Beta-Cell Function (HOMA-β), Triglyceride-Glucose Index (TyG), Triglyceride-Glucose Body Mass Index (TyG-BMI), Triglyceride-Glucose Waist-to-Height Ratio (TyG-WtHR), Triglyceride-Glucose Waist Circumference (TyG-WC), Metabolic Score for Insulin Resistance (METS-IR), and Visceral Adiposity Index (VAI), on BMD to clarify the possible role of insulin resistance in the determination of bone health.

## Materials and methods

The PRISMA statement guidelines were followed during the conduct of this meta-analysis (21). This study adhered to a predetermined process outlined in the prospective register of systematic reviews (PROSPERO) (CRD42024512770).

### Search strategy and screening

We conducted a search on electronic databases, including PubMed, Embase, Web of Science, and Scopus, until July 2024. The search utilized the following terms or relevant Medical Subject Headings (MeSH): (“visceral adiposity index” [Title/Abstract] OR “VAI” [Title/Abstract] OR “lipid accumulation product” [Title/Abstract] OR “LAP” [Title/Abstract] OR “triglyceride glucose index” [Title/Abstract] OR “TyG” [Title/Abstract] OR “triglyceride-glucose index” [Title/Abstract] OR “TyG-body mass index” [Title/Abstract] OR “TyG-BMI” [Title/Abstract] OR “TyG-waist circumference” [Title/Abstract] OR “TyG-WC” [Title/Abstract] OR “Homeostatic Model Assessment for Insulin Resistance” [Title/Abstract] OR “HOMA-IR” [Title/Abstract] OR “Metabolic Syndrome Insulin Resistance” [Title/Abstract] OR “MetS-IR” [Title/Abstract] OR “Lipoprotein Insulin Resistance Index” [Title/Abstract] OR “LP-IR” [Title/Abstract] OR “TyG-NC” [Title/Abstract] OR “TyG-NHtR” [Title/Abstract] OR “triglycerides to HDL cholesterol ratio” [Title/Abstract] OR “TG/HDL-C” [Title/Abstract] OR “Adipose insulin resistance index” [Title/Abstract] OR “Adipo-IR” [Title/Abstract] OR “lipid indices” [Title/Abstract] OR “Insulin Resistance index” [Title/Abstract] OR “Insulin Resistance indices” [Title/Abstract]) AND (“bone density”[MeSH] OR “fractures, bone”[MeSH] OR “osteoporosis”[MeSH] OR “osteoporosis”[Title/Abstract] OR “osteoporotic”[Title/Abstract] OR “osteoporoses”[Title/Abstract] OR “bone loss”[Title/Abstract] OR “fracture”[Title/Abstract] OR “bone demineralisation”[Title/Abstract] OR “bone demineralization”[Title/Abstract] OR “metabolic bone disease*”[Title/Abstract] OR “osteopenia”[Title/Abstract] OR “osteopenic”[Title/Abstract] OR “osteopaenia”[Title/Abstract] OR “osteopaenic”[Title/Abstract] OR “bone density”[Title/Abstract] OR “bone deterioration”[Title/Abstract] OR “bone mass density”[Title/Abstract] OR “bone mineral density”[Title/Abstract] OR “BMD”[Title/Abstract]). Further articles were screened for eligibility by referencing the included studies. Rayyan, a free online web tool for systematic reviewing, was used to screen the studies. It is accessible at https://www.rayyan.ai. Two reviewers (A.G.R. and A.A.) independently assessed each study and thoroughly examined the entire text to remove any duplicate materials. Studies meeting the inclusion-exclusion criteria were chosen. The third author (A.H.H.) conducted consensus meetings to address any potential disagreements among reviewers.

### Inclusion and exclusion criteria

Population (P): Adult participants; Exposure (E): Studies assessing insulin resistance surrogate indices (e.g., HOMA-IR, TyG, VAI).; Comparison (C): Individuals with different levels of insulin resistance surrogate indices; Outcomes (O): BMD of different areas when beta coefficient was reported by multivariate analysis; Type of Design (T): Observational studies. Here are the formulas illustrating the definitions for the insulin surrogate indices.


$$\text{HOMA}‐\text{IR}=\frac{\text{Fasting}\;\text{Insulin}\;(\mu \text{U}/\text{mL})\times \text{Fasting Plasma Glucose}\;(\text{mmol}/\text{L})}{22.5}$$

$$\text{HOMA}‐\text{B}=\frac{20\times \text{Fasting}\;\text{Insulin}\;\left(\mu \text{U}/\text{mL}\right)}{\text{Fasting}\;\text{Plasma}\;\text{Glucose}\;(\text{mmol}/\text{L})-3.5}$$

For men:


$$\begin{array}{c} \text{VAI}=(\frac{\text{WC}\;(\text{cm})}{39.68+(1.88\times \text{BMI})})\times \frac{\text{TG}\;(\text{mmol}/\text{L})}{1.03}\\ \;\;\;\times \frac{1.31}{\text{HDL}‐\text{C}\;(\text{mmol}/\text{L})} \end{array}$$

For women:


$$\begin{array}{c} \text{VAI}=(\frac{\text{WC}\;(\text{cm})}{36.58+(1.89\times \text{BMI})})\times \frac{\text{TG}\;(\text{mmol}/\text{L})}{0.81}\\ \;\;\;\times \frac{1.52}{\text{HDL}‐\text{C}\;(\text{mmol}/\text{L})} \end{array}$$

TyG=ln (TG (mg/dL)×FPG (mg/dL)÷2)

$$\text{TyG}‐\text{BMI}=\text{TyG}\times \text{BMI}\;\left(\text{kg}/{\text{m}}^{2}\right)$$

TyG‐WtHR=TyG×WtHR

TyG‐WC=TyG×WC (cm)

$$\begin{array}{c} \text{METS‐IR}=\text{ln}\;\;(\text{FPG}\;(\text{mg}/\text{dL})\times \text{TG}\;\left(\text{mg}/\text{dL}\right)\\ \times \text{BMI}\;(\text{kg}/{\text{m}}^{2}))÷\text{HDL‐C}\;(\text{mg}/\text{dL}) \end{array}$$

Exclusion criteria were studies that involved patients with diabetes, studies without adjustment for covariates such as multivariable logistic regression analysis, non-English studies, studies that did not report BMD values (T score or Z score), FRAX or osteoporosis (low T score +/- fracture), as well as case reports, reviews, editorials, commentaries, and conference abstracts that lacked original research data or detailed methodologies.

### Data extraction and quality assessment

After conducting a full-text screening, two researchers (S.M.A., Y.T.) separately entered the data into an existing Excel spreadsheet. This document included two categories of data (1): demographic details like journal, authors, publication year, study location, design, sample size, gender, patient age, IR index, and outcomes; and (2) specific results such as covariates, beta coefficient, insulin resistance indices (HOMA-IR, HOMA-β, VAI, TyG, TyG-BMI, TyG-WTHR, TyG-WC, and METS-IR) levels, and BMD of various anatomical sites. The third reviewer (A.H.H.) assessed the conflicts.

Two authors (N.Z., A.A.) individually assessed the studies’ quality using the critical appraisal checklists developed by the Joanna Briggs Institute (JBI) for cohort studies (22). The JBI critical evaluation checklist consists of eleven components for cohort studies and eight for cross-sectional studies. The checklist assesses specific research topics to detect possible bias risks and offers direct binary answers. If the answer was yes, the question received a score of 1. Responses that were negative, ambiguous, or irrelevant were assigned a score of 0 (23). Scheduled meetings were arranged in order to come to a common agreement and settle any disagreements.

## Results

### Study selection

**Figure 1:**
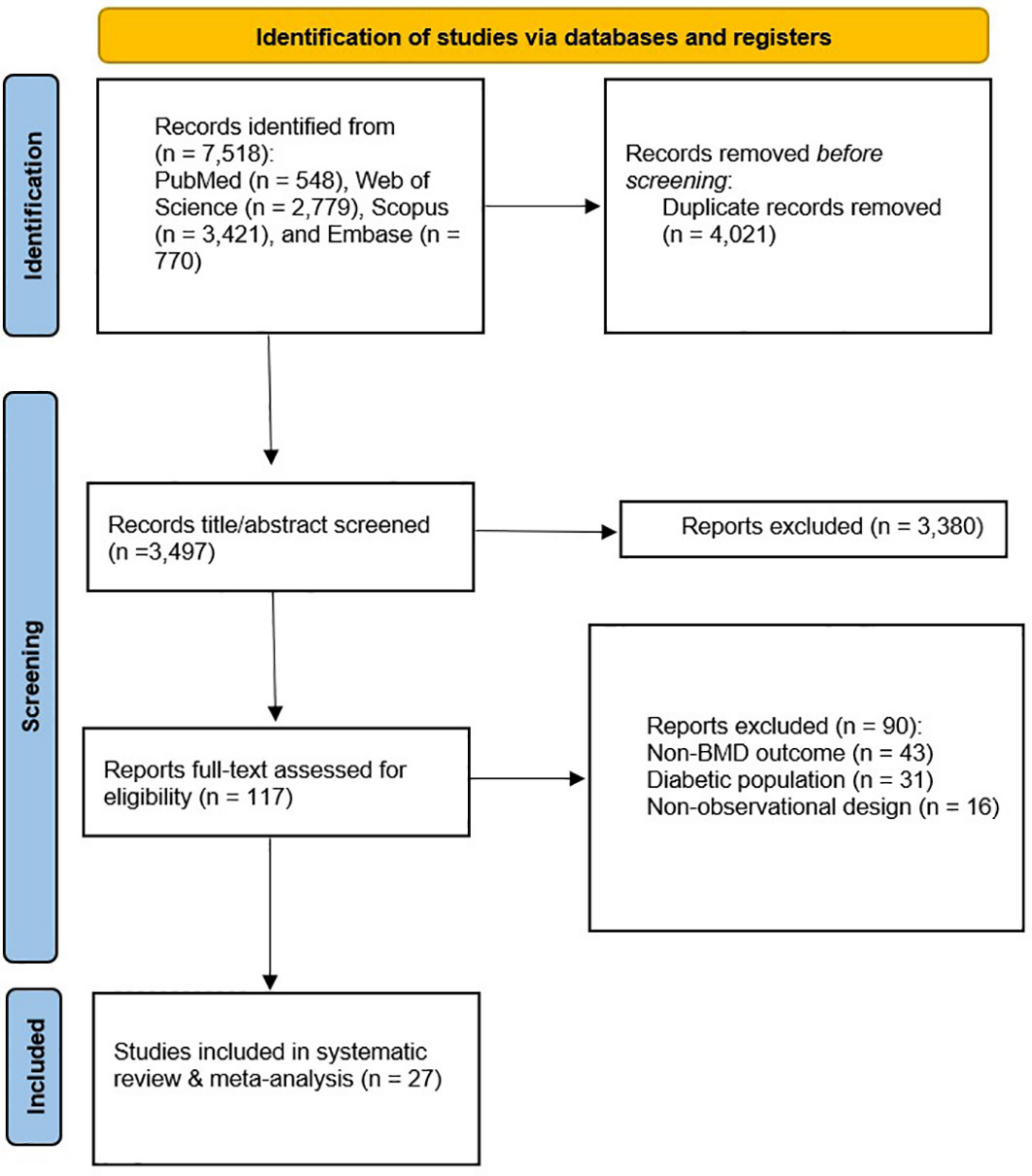
PRISMA flow diagram.

The initial systematic search of databases, including PubMed, Web of Science, Scopus, and Embase, identified 7,518 studies. After removing duplicates (n = 4,021), 3,497 records proceeded to title and abstract screening, of which 3,380 studies were excluded as irrelevant. A full-text assessment was conducted for the remaining 117 studies. Twenty-seven of these studies were deemed eligible for systematic review (**Figure 1**).

### Baseline characteristics and quality assessment

This systematic review included studies conducted across various geographic regions, comprising the USA (5, 20, 24–32), China (33–37), Korea (19, 38–41), Brazil (42, 43), Japan (44), Singapore (45), Serbia (46) and Spain (47). The studies utilized cohort (20, 36, 44, 45) and cross-sectional (5, 19, 24–35, 37–43, 46, 47) methodologies. Data for 71,525 participants (52.1% female) were analyzed. The mean age of our population ranged from 30.3 (19) to 73.6 (20). Different indices, such as HOMA-IR, HOMA-β, VAI, TyG, TyG-BMI, TyG-WTHR, TyG-WC, and METS-IR, were used to indicate insulin resistance. Various outcome measures and additional information are summarized in **Table 1**.

**Table 1:** Baseline characteristics of the included studies.

Author	Year	Country	Study Design	Analytic sample	Age (mean±SD)	Female %	IR index	Outcomes
Kim	2013	South Korea	Cross-sectional	14485	45.4±0.3	54.03	HOMA-IR	BMD (Lumbar spine, Total hip, and Femoral neck)
Shin	2014	South Korea	Cross-sectional	3113	49.7±0.6	0	HOMA-IR	BMD (Whole body, Lumbar spine, and Femoral neck)
Srikanthan	2014	USA	Cross-sectional	717	56.8±11.3	51.6	HOMA-IR	BMD (Lumbar spine and Femoral neck)
Shanbhogue	2016	USA	Cross-sectional	146	60.3±2.7	100	HOMA-IR	BMD (Lumbar spine and Total hip)
Choo	2017	South Korea	Cross-sectional	2750	30.3±0.2	56.07	HOMA-IR	BMD (Total hip, Femoral neck, Lumbar spine, Femoral trochanter, and Femoral intertrochanter)
Iki	2012	Japan	Prospective Cohort	1683	72.9±5.2	0	HOMA-IR, HOMA-β	BMD (Lumbar spine)
Seoung	2018	South Korea	Cross-sectional	137	55.6±5.8	100	HOMA-IR	BMD (Lumbar spine and Femoral neck)
Kalimeri	2018	China-Singapore	Cohort	96	60.7±4.2	100	HOMA-IR	BMD (Lumbar spine, Total hip and Femoral neck)
Napoli	2019	USA	Prospective Cohort	2398	73.6±2.9	53	HOMA-IR	BMD (Total hip)
Yang	2019	China	Cross-sectional	892	55±1.1	100	HOMA-IR	BMD (Lumbar spine and Femoral neck)
de Araújo	2020	Brazil	Cross-sectional	56	47±14	64.28	HOMA-IR	BMD (Lumbar spine, Total hip, and Femoral neck, Radius)
Wang	2020	China	Cross-sectional	2122	45.1±15	59.94	HOMA-IR	BMD (Forearm)
Campillo-Sánchez	2020	Spain	Cross-sectional	381	62±8.6	100	HOMA-IR	BMD (Total hip and Femoral neck)
Yoon	2021	Korea	Cross-sectional	4810	62.7±8.7	46.94	HOMA-IR, TyG	BMD (Whole body, Lumbar spine, and Femoral neck, Total hip)
Giudici	2021	Brazil	Cross-sectional	298	57.5±8.8	55.7	HOMA-IR, HOMA-β	BMD (Whole body, Lumbar spine, and Femoral neck)
Ye	2023	China	Cross-sectional	437	53.5±1.2	100	HOMA-IR	BMD (Femoral neck)
Sun	2023	USA	Cross-sectional	3341	69.8±6.9	47.4	VAI	BMD (Total femur, Femur neck, Trochanter, Intertrochanter)
Pu	2023	USA	Cross-sectional	1114	58.6±12.2	50.9	METS-IR	BMD (Lumbar spine and Total femur)
Chen	2023	USA	Cross-sectional	6257	50.2±15.5	50.1	VAI	BMD (Total femur)
Zhan	2023	USA	Cross-sectional	3646	37.4±11.2	46.4	TyG	BMD (Lumbar, Whole body)
Xuan	2024	USA	Cross-sectional	1182	60.3±8.2	0	TyG-BMI	BMD (Femoral neck)
Tian N	2024	USA	Cross-sectional	5456	30.3±13.5	44.35	TyG, TyG-BMI, TyG-WHTR, TyG-WC	BMD (Whole body)
Chen	2024	USA	Cross-sectional	1844	60.7±0.3	37	TyG	BMD (Femoral neck)
Tian C	2024	USA	Cross-sectional	6501	51.2±16.9	49.3	TyG-BMI	BMD (Total femur, Femur neck, Trochanter, Intertrochanter)
Wen	2022	China	Prospective cohort	832	59.3±7.8	43.0	TyG-BMI	BMD (Total hip, Lumbar spine, Femoral neck)
Shao	2024	China	Cross-sectional	6769	47.4±17.5	49.0	METS-IR	BMD (Total femur, Femoral neck, Total spine)
Sretenović	2021	Serbia	Cross-sectional	62	71.2±4.8	100	HOMA-IR	BMD, T score and Z score (Hip, Lumbar spine)

25(OH)D, 25-Hydroxyvitamin D; 25(OH)D2, 25-Hydroxyvitamin D2; 25(OH)D3, 25-Hydroxyvitamin D3; ALP, Alkaline Phosphatase; ALT, Alanine Transaminase; AST, Aspartate Transaminase; BFM, Body Fat Mass; BMD, Bone Mineral Density; BMI, Body Mass Index; BUN, Blood Urea Nitrogen; CPK, Creatine Phosphokinase; CRP, C-Reactive Protein; CVD, Cardiovascular Disease; DBP, Diastolic Blood Pressure; eGFR, Estimated Glomerular Filtration Rate; FMI, Fat Mass Index; FPG, Fasting Plasma Glucose; FSH, Follicle-Stimulating Hormone; HDL-C, High-Density Lipoprotein Cholesterol; HOMA-IR, Homeostatic Model Assessment for Insulin Resistance; HOMA-β, Homeostatic Model Assessment for Beta-cell Function; Hs-CRP, High-sensitivity C-Reactive Protein; IFG, Impaired Fasting Glucose; LMI, Lean Mass Index; LDL-C, Low-Density Lipoprotein Cholesterol; MetS-IR, Metabolic Syndrome Insulin Resistance; PIR, Poverty Income Ratio; SBP, Systolic Blood Pressure; SCr, Serum Creatinine; SMM, Skeletal Muscle Mass; SUA, Serum Uric Acid; TC, Total Cholesterol; TG, Triglycerides; TBS, Trabecular Bone Score; tOC, Total Osteocalcin; TyG, Triglyceride Glucose Index; TyG-BMI, TyG-Body Mass Index; TyG-WC, TyG-Waist Circumference; TyG-WHtR, TyG-Waist-to-Height Ratio; UA, Uric Acid; VAI, Visceral Adiposity Index; VFA, Visceral Fat Area; WC, Waist Circumference; WHR, Waist-to-Hip Ratio; ucOC, Undercarboxylated Osteocalcin; anti-TPO, Anti-Thyroid Peroxidase Antibody; TSH, Thyroid-Stimulating Hormone; Ft4, Free Thyroxine; anti-Tg, Anti-Thyroglobulin Antibody; IGF, Insulin-like Growth Factor; PTH, Parathyroid Hormone.

The quality assessment of cross-sectional studies in this review indicated a uniformly high standard, with all studies meeting each of the eight JBI quality criteria. The cohort studies also demonstrated high quality, with most criteria consistently met across studies. However, there were some limitations in addressing incomplete follow-up in two of the cohort studies (44, 45), which did not employ strategies to address this issue. Despite these minor limitations, the overall methodological quality of the included studies was robust (**Supplementary Tables S1**, **S2**).

### HOMA-IR and bone mineral density

A number of 17 studies reported the association between HOMA-IR and BMD (5, 19, 20, 24, 33–35, 38–47). Several results of studies reported that elevated levels of HOMA-IR were inversely associated with BMD (5, 19, 34, 38, 39, 41, 45), with the adjusted regression coefficient ranging from -1.11 (38) to -0.021 (39). As for specific sites of BMD, higher levels of HOMA-IR had a negative effect on whole-body BMD, with the adjusted regression coefficient ranging from -0.041 (41) to -0.025 (39), femoral neck BMD [from -1.09 (38) to -0.021 (39)], and lumbar BMD [from β = -0.49 (38) to β = -0.084 (19)]. Numerous results showed that the level of HOMA-IR does not statistically correlate with BMD (5, 19, 20, 24, 38–47). Kim et al., with a sample size of 14,485, revealed that in premenopausal women, no association was found between the HOMA-IR index and lumbar spine BMD (β = -0.16, P = 0.352). Lumbar spine, total hip, and femoral neck BMD were not correlated with the HOMA-IR level in postmenopausal women (β = 0.2, P = 0.482; β = 0.03, P = 0.940; and β = -0.45, P = 0.409) (38). Yoon et al., with a sample size of 4,810, reported that HOMA-IR in men’s lumbar spine (β = -0.014, P = 0.499) and total hip (β = -0.021, P = 0.344), as well as in women’s lumbar spine (β = 0.006, P = 0.813), femoral neck (β = -0.043, P = 0.115), total hip (β = 0.013, P = 0.620), whole body (β = -0.026, P = 0.260) BMD, were not associated with HOMA-IR levels (41). In contrast, Yang et al. identified a direct correlation between HOMA-IR and BMD of the lumbar spine and femoral neck (β = 0.103, P = 0.002; β = 0.091, P = 0.009) (33). Likewise, Ye et al. found that greater insulin resistance was associated with increased femoral neck BMD in nondiabetic postmenopausal women (β [95%CI] = 0.025 [0.003, 0.047], P = 0.026) (35).

### TyG and bone mineral density

Four studies reported on the relationship between TyG index and BMD (28, 30, 32, 41). Yoon et al. found that an inverse association existed between TyG index and femoral neck, total hip, and whole-body BMD in non-diabetic men (β = -0.085, P < 0.001; β = -0.046, P = 0.037; β = -0.098, P < 0.001) and femoral neck and whole-body BMD in women (β = -0.071, P = 0.008; β = -0.065, P = 0.005), but no association was observed in the total hip area BMD of women (β = -0.003, P = 0.911). Lumbar spine BMD was found to have no relationship with TyG index in men (β = -0.028, P = 0.168) or women (β = 0.016, P = 0.500) (41). According to Zhan et al., an inverse correlation existed between TyG index and BMD of the lumbar spine and whole body (β [95%CI] = -0.008 [-0.017, 0], β [95%CI] = -0.007 [-0.012, -0.001]) (28). Conversely, Tian N et al. found a positive correlation between whole-body BMD and TyG (β [95%CI] = 0.0124 [0.001, 0.024]) (30). Furthermore, Chen et al. found no association between femoral neck BMD and TyG index (32). Therefore, most of the literature suggests that a higher TyG index, reflecting higher insulin resistance, is generally associated with lower BMD.

### VAI and bone mineral density

Two studies reported on VAI and BMD (25, 27). There was a positive association between VAI and BMD of total femur, femoral neck, trochanter, and intertrochanter, Sun et al. reported (β [95%CI] = 0.006 [0.004, 0.009], P < 0.001; β [95%CI] = 0.004 [0.002, 0.006], P = 0.001; β [95%CI] = 0.005 [0.003, 0.007], P < 0.001; β [95%CI] = 0.007 [0.004, 0.010], P < 0.001) (27). Chen et al. displayed a substantially positive association between femoral BMD and VAI (β [95%CI] = 0.016 [0.014, 0.019], P < 0.001) (25). Hence, a greater VAI index, indicating increased insulin resistance, was generally linked to higher BMD.

### Other insulin surrogate indices and bone mineral density

HOMA-β (43, 44), TyG-BMI (29–31, 36), METS-IR (26), TyG-WTHR (30), and TyG-WC (30) were reported in very few studies. Giudici et al. reported on HOMA-β and BMD of the total body, lumbar spine, and femur (β = -0.006, P = 0.631; β = 0.071, P = 0.206; β = -0.021, P = 0.089), and found no statistically significant association (43). Similarly, Iki et al. found no correlation between HOMA-β and lumbar spine BMD (β = 0.043, P = 0.0948) (44). Therefore, it can be deduced that no association exists between HOMA-β and BMD.

As for TyG-BMI, Xuan et al. assessed insulin resistance by TyG-BMI, which demonstrated a positive association between TyG-BMI and femoral neck BMD as well (β [95%CI] = 0.058 [0.045, 0.072], P < 0.001) (29), similar to Tian N et al. assessing whole-body BMD and TyG-BMI (β [95%CI] = 0.0004 [0.0003, 0.0004], P <0.0001) (30) and Tian C et al. assessing total femur, femur neck, trochanter, intertrochanter BMD and TyG-BMI (β [95%CI] = 0.002 [0.002, 0.002], P<0.00001; β [95%CI] = 0.001 [0.001, 0.002], P<0.00001; β [95%CI] = 0.001 [0.001, 0.001], P<0.00001; β [95%CI] = 0.002 [0.002, 0.002], P<0.00001) (31). Likewise, Wen et al. indicated positive correlation between TyG-BMI and femoral neck, lumbar spine and total hip BMD in both male (respectively β = 0.224, 0.185, 0.271; p < 0.001) and female (respectively β = 0.279, 0.192, 0.415; p < 0.001) (36).

Regarding METS-IR, Pu et al. illustrated a direct correlation between elevated levels of METS-IR, indicating higher insulin resistance, and increased total femoral and lumbar spine BMD (β [95%CI] = 0.005 [0.004, 0.006]; β = 0.005 [0.004, 0.006]) (26). Similarly, Shao et al. found positive association between METS-IR and BMD of total femur, femoral neck and total spine (β[95%CI] = 0.060 [0.057, 0.064]; β[95%CI] = 0.049 [0.045, 0.052]; β[95%CI] = 0.040 [0.036, 0.044]; P<0.001) (37).

Lastly, a positive association was seen between whole-body BMD and TyG-WTHR (β[95%CI] = 0.012 [0.008, 0.016], P<0.0001) and TyG-WC (β[95%CI] = 0.0001 [0.0001, 0.0001], P<0.0001) (30). Summarized information regarding the qualitative synthesis of the included data is visibale in **Table 2**.

**Table 2:** Summary of main findings.

Study	IR index	Adjustment Covariates	Adjusted β	Conclusion
Giudici 2021	HOMA-IR, HOMA-β	Age, Sex, LMI, FMI, and 25(OH)D	Whole body BMD and HOMA-IR = -0.011 Lumbar spine BMD and HOMA-IR= -0.018 Femoral BMD and HOMA-IR= -0.002 Whole body BMD and HOMA-β = -0.006 Lumbar spine BMD and HOMA-β = 0.071 Femoral BMD and HOMA-β = -0.021	No association between HOMA-IR/HOMA-β and BMD
Campillo-Sánchez 2020	HOMA-IR	Past And Present Medication, Lifestyle Factors, Age, BMI, the Presence of Osteoporosis and Osteoporosis Risk Factors.	Femoral neck BMD and HOMA-IR= 0.224 Total hip BMD and HOMA-IR= 0.225	No association between HOMA-IR and BMD
Kim 2013	HOMA-IR	Age, BMI, Smoking, Alcohol, and Regular Exercise	Men Lumbar spine BMD and HOMA-IR= -0.49 Femoral neck BMD and HOMA-IR= -1.09 Total hip BMD and HOMA-IR= -1.11 Premenopausal women Lumbar spine BMD and HOMA-IR= -0.16 Femoral neck BMD and HOMA-IR= -0.33 Total hip BMD and HOMA-IR= -0.37 Postmenopausal Lumbar spine BMD and HOMA-IR= 0.20 Femoral neck BMD and HOMA-IR= -0.45 Total hip BMD and HOMA-IR= 0.03	In men, negative association between HOMA-IR and BMD. In premenopausal women negative association between HOMA-IR and femoral neck and total hip, no association with lumbar spine BMD. In postmenopausal women, no association between HOMA-IR and BMD.
Shin 2014	HOMA-IR	Age, Weight, Height, Smoking, Alcohol, Income, Physical Activity, Calcium Intake, 25(OH)D, Diabetes, Percent Fat Mass, TC, HDL-C, and TG	Whole-Body BMD and HOMA-IR= -0.03 Femoral Neck BMD and HOMA-IR= -0.02 Lumbar Spine BMD and HOMA-IR= -0.01	Negative association between HOMA-IR and whole body and femoral neck BMD, but no association with lumbar spine BMD
Srikanthan 2014	HOMA-IR	Age, Sex, Race/Ethnicity, Menopause Transition Stage in Women, And Study Site	Lumbar spine BMD and HOMA-IR=-0.09 (-0.07, -0.02) Femoral neck BMD and HOMA-IR= -0.05 (-0.13, 0.027)	Negative association between lumbar spine BMD and HOMA-IR, no association between femoral neck BMD and HOMA-IR
Shanbhogue 2016	HOMA-IR	Weight, Time Since Menopause, Tobacco, Alcohol, Physical Activity, Prior Use Of Osteoporosis Medications, Systemic HRT, or Glucocorticoids.	Lumbar spine BMD and HOMA-IR= 0.01 (-0.17, 0.19) Total hip BMD and HOMA-IR = 0.166 (-0.01, 0.34)	No association between BMD and HOMA-IR
Choo (men) 2017	HOMA-IR	Gender, Age, Height, Weight, BFM, SBP, DBP, TC, TG, HDL-C, LDL-C, 25(OH)D, Smoking, Alcohol, Physical Activity, Education Level, Household Income, Use of Oral Contraceptives, and Age at Menarche in Females	Total hip BMD and HOMA-IR= -0.07 Femoral neck BMD and HOMA-IR= -0.1 Lumbar spine BMD and HOMA-IR= -0.08 Femoral trochanter BMD and HOMA-IR= -0.066 Femoral intertrochanter BMD and HOMA-IR= -0.054	Negative association between HOMA-IR and BMD
Choo 2017 (women)	HOMA-IR	Gender, Age, Height, Weight, BFM, SBP, DBP, TC, TG, HDL-C, LDL-C, 25(OH)D, Smoking, Alcohol, Physical Activity, Education Level, Household Income, Use of Oral Contraceptives, and Age at Menarche in Females	Total hip BMD and HOMA-IR= -0.044 Femoral neck BMD and HOMA-IR= -0.051 Lumbar spine BMD and HOMA-IR= -0.035 Femoral trochanter BMD and HOMA-IR= -0.04 Femoral intertrochanter BMD and HOMA-IR= -0.03	Negative association between femoral neck BMD and HOMA-IR, no association between total hip, femoral trochanter and intertrochanter BMD and HOMA-IR
Iki 2012	HOMA-IR, HOMA-β	Age, BMI and TBS	Lumbar spine BMD and HOMA-IR= 0.013 Lumbar spine BMD and HOMA-β= 0.043	No association between HOMA-IR, HOMA-β and BMD
Seoung 2018	HOMA-IR	Age, Years Since Menopause, BMI, Smoking, Alcohol, WHR, VFA, BFM, SMM, TC, TG, HDL C, LDL-C, FPG, Hs-CRP, Adiponectin, Leptin, tOC, ucOC, Hypertension, And Lipid-Lowering Therapy	Lumbar spine BMD and HOMA-IR= 0.012 (-0.021, 0.024) Femoral neck BMD and HOMA-IR= 0.059 (-0.021, 0.038)	No association between BMD and HOMA-IR
Kalimeri 2018	HOMA-IR	Lean Body Mass, Age	Femoral neck BMD and HOMA-IR= -0.064 Total hip BMD and HOMA-IR= -0.096 Lumbar spine BMD and HOMA-IR= -0.199	Negative association between lumbar spine BMD and HOMA-IR, no association for femoral neck and total hip BMD and HOMA-IR
Napoli 2019	HOMA-IR	Age, Sex, Race, eGFR, Clinic Site, BMI	Total hip BMD and HOMA-IR= Reference Total hip BMD and HOMA-IR= -0.006 Total hip BMD and HOMA-IR= 0.004 Total hip BMD and HOMA-IR= 0.007	No association between HOMA-IR and BMD
Yang 2019	HOMA-IR	First-degree FHD, Age, SUA, BMI, Menopausal Period, eGFR	Lumbar spine BMD and HOMA-IR= 0.103 Femoral neck BMD and HOMA-IR= 0.091	Positive correlation between HOMA-IR and BMD
de Araújo 2020	HOMA-IR	Age and BMI	L1-L4 BMD and HOMA-IR= 0.013 Total hip BMD and HOMA-IR= 0.018 Femoral neck BMD and HOMA-IR= 0.01 1/3 radius BMD and HOMA-IR= 0.01 L3 BMD and HOMA-IR= 0.038	No association between HOMA-IR and BMD
Zhan 2023	TyG	Age, Gender, Race, Education, Moderate Recreational Activities, Diabetes, UA, Calcium, Phosphorus, WC, LDL-C, Smoking, 25(OH)D, Antihyperlipidemic Agents	Lumbar spine BMD and TyG= -0.01(-0.02, 0) Whole-body BMD and TyG= -0.01(-0.01, -0.001) Subtotal BMD and TyG= -0.005(-0.011, 0)	Negative association between TyG index and BMD
Xuan 2024	TyG-BMI	Age, Race/Ethnicity, Education, Marital Status, Drinking, Smoking, SBP, DBP, TC, HDL-C, LDL-C, Family of Osteoporosis, Physical Activity, PIR, Calcium, Phosphorus, 25(OH)D3	Femoral neck BMD and TyG-BMI = 0.058 (0.045, 0.072)	Positive association between TyG-BMI and BMD
Chen 2023	VAI	Race, Gender, Age, Education Level, Smoked At Least 100 Cigarettes, Moderate Activities, Diabetes, Family PIR, BUN, AST, ALP, ALT, Cr, Phosphorus, TC, Calcium, and Total Protein	Femoral BMD and VAI= 0.016	Positive association between VAI and BMD
Ye 2023	HOMA-IR	Age, FSH, CRP, And IFG, Physical Activity, Drinking and Smoking, BMI	Femoral neck BMD and HOMA-IR= 0.025 (0.003, 0.047)	Positive association between HOMA-IR and BMD
Yoon 2021 (men)	TyG, HOMA-IR	Age, BMI, 25(OH)D, Physical Activity, Smoking, and Drinking	Lumbar spine BMD and TyG= -0.028 Femoral neck BMD and TyG= -0.085 Total hip BMD and TyG= -0.046 Whole body BMD and TyG= -0.098	In men, negative association between TyG and femoral neck, total hip, and whole-body BMD and HOMA-IR and femoral neck and whole body. No association for others.
Yoon 2021 (women)	TyG, HOMA-IR	Age, BMI, 25(OH)D, Physical Activity, Smoking, and Drinking	Men Lumbar spine BMD and TyG= -0.028 Femoral neck BMD and TyG= -0.085 Total hip BMD and TyG= -0.046 Whole body BMD and TyG= -0.098 Women Lumbar spine BMD and TyG= 0.016 Femoral neck BMD and TyG= -0.071 Total hip BMD and TyG= -0.003 Whole body BMD and TyG= -0.065 Men Lumbar spine BMD and HOMA-IR= -0.014 Femoral neck BMD and HOMA-IR= -0.051 Total hip BMD and HOMA-IR= -0.021 Whole body BMD and HOMA-IR= -0.041 Women Lumbar spine BMD and HOMA-IR= 0.006 Femoral neck BMD and HOMA-IR= -0.043 Total hip BMD and HOMA-IR= 0.013 Whole body BMD and TyG= -0.026	In women, negative association between TyG and femoral neck and whole-body BMD, No association for others.
Sun 2023	VAI	Gender, Age, Race, Education Level, Marital Status, PIR, Smoking, Work Activity, BUN, Calcium, Phosphorus, and SUA	Total femur BMD and VAI= 0.006 (0.004-0.009) Femur neck BMD and VAI= 0.004 (0.002-0.006) Trochanter BMD and VAI= 0.005 (0.003-0.007) Intertrochanter BMD and VAI= 0.007 (0.004-0.010)	Positive association between VAI and BMD
Pu 2023	METS-IR	Age, Race, Education, Marital Status, PIR, Smoking, Alcohol, Hypertension, Calcium, 25(OH)D, TC, LDL-C, SCr, SUA, and BUN	Total femoral BMD and METS-IR= 0.005(0.004, 0.006) Lumbar spine BMD and METS-IR= 0.005(0.004, 0.006)	Positive association between METS-IR and BMD
Wang 2020	HOMA-IR	Age, BMI, Height, and Weight	HOMA-IR and forearm BMD= -0.15	Negative association of HOMA-IR and BMD
Tian N 2024	TyG, TyG-BMI, TyG-WHtR, TyG-WC	Age, Gender, Race, Education Level, Family PIR, ALP, BUN, CPK, Creatinine, Phosphorus, Calcium, UA, Total Bilirubin, Glycohemoglobin, TC, HDL-C, LDL-C and 25OHD2, 25OHD3	Whole-body BMD and TyG = 0.0124 (0.001, 0.024) Whole-body BMD and TyG-BMI = 0.0004 (0.0003, 0.0004) Whole-body BMD and TyG-WHTR = 0.012 (0.008, 0.016) Whole-body BMD and TyG-WC = 0.0001 (0.0001, 0.0001)	Positive association of TyG, TyG-BMI, TyG-WHTR, TyG-WC and BMD
Chen 2024	TyG	Age, BMI, Race, CVD history, FPG, fasting insulin, and 25(OH)D3	Men Femur neck BMD and TyG = −0.0003 (−0.02-0.01) Women Femur neck BMD and TyG = −0.002 (−0.03-0.02)	No association between TyG and BMD
Tian C 2024	TyG-BMI	Age, Sex, Race, PIR, Education Attainment, Central Obesity, Calcium, Phosphorus, ALP, ALT, AST, 25(OH)D3, Physical Activity Level, Use of Glucocorticoid, Fracture History, Smoked At Least 100 Cigarettes in Life, Had At Least 12 Alcohol Drinks Past 1 Year, Hypertension	Total femur BMD and TyG-BMI= 0.002 (0.002, 0.002) Femur neck BMD and TyG-BMI= 0.001 (0.001, 0.002) Trochanter BMD and TyG-BMI= 0.001 (0.001, 0.001) Intertrochanter BMD and TyG-BMI= 0.002 (0.002, 0.002)	Positive association between TyG-BMI and BMD
Wen 2022	TyG-BMI	Age, Smoking, Drinking, Previous Fracture, Parental Hip Fracture.	Men Femur neck BMD and TyG-BMI= 0.224 Lumbar spine BMD and TyG-BMI= 0.185 Total hip BMD and TyG-BMI= 0.271 Women Femur neck BMD and TyG-BMI= 0.279 Lumbar spine BMD and TyG-BMI= 0.192 Total hip BMD and TyG-BMI= 0.415	Positive association between TyG-BMI and BMD
Shao 2024	METS-IR	Gender, Age, Race, Education, Marital Status, Smoking, Activity, Hypertension, Diabetes, Calcium, UA, BUN, TC, Insulin or Glucose-Lowering Drugs, Prednisone or Cortisone, History of Osteoporosis	Total femur BMD Q1 = Reference Q2 = 0.052 (0.041, 0.062) Q3 = 0.094 (0.084, 0.105) Q4 = 0.150 (0.140, 0.161) Femur neck BMD Q1 = Reference Q2 = 0.027 (0.016, 0.037) Q3 = 0.061 (0.050, 0.071) Q4 = 0.115 (0.105, 0.126) Total spine BMD Q1 = Reference Q2 = 0.051 (0.041, 0.062) Q3 = 0.064 (0.053, 0.074) Q4 = 0.108 (0.098, 0.119)	Positive association between METS-IR and BMD
Sretenović 2021	HOMA-IR	FPG, Vit D, Somatropin, anti-TPO, TSH, Ft4, anti-Tg, IGF, PTH	Hip BMD and HOMA-IR = 0.036 (-6.036, 7.239) Lumbar spine BMD and HOMA-IR = 0.032 (-5.719, 6.807) Femoral neck BMD and HOMA-IR = 0.311 (-.093, 8.762) Hip T-score and HOMA-IR = 0.066 (-2.732, 3.005) Spine T-score and HOMA-IR = 0.387 (-2.156, 3.332) Hip Z-score and HOMA-IR = 0.274 (-2.218, 3.373) Spine Z-score and HOMA-IR = -0.154 (-3.011, 2.544)	No association between HOMA-IR and hip and spine BMD

25(OH)D, 25-Hydroxyvitamin D; 25(OH)D2, 25-Hydroxyvitamin D2; 25(OH)D3, 25-Hydroxyvitamin D3; ALP, Alkaline Phosphatase; ALT, Alanine Transaminase; AST, Aspartate Transaminase; BFM, Body Fat Mass; BMD, Bone Mineral Density; BMI, Body Mass Index; BUN, Blood Urea Nitrogen; CPK, Creatine Phosphokinase; CRP, C-Reactive Protein; DBP, Diastolic Blood Pressure; eGFR, Estimated Glomerular Filtration Rate; FMI, Fat Mass Index; FPG, Fasting Plasma Glucose; FSH, Follicle-Stimulating Hormone; HDL-C, High-Density Lipoprotein Cholesterol; HOMA-IR, Homeostatic Model Assessment for Insulin Resistance; HOMA-β, Homeostatic Model Assessment for Beta-cell Function; Hs-CRP, High-sensitivity C-Reactive Protein; IFG, Impaired Fasting Glucose; LMI, Lean Mass Index; LDL-C, Low-Density Lipoprotein Cholesterol; MetS-IR, Metabolic Syndrome Insulin Resistance; PIR, Poverty Income Ratio; SBP, Systolic Blood Pressure; SCr, Serum Creatinine; SMM, Skeletal Muscle Mass; SUA, Serum Uric Acid; TC, Total Cholesterol; TG, Triglycerides; TBS, Trabecular Bone Score; tOC, Total Osteocalcin; TyG, Triglyceride Glucose Index; TyG-BMI, TyG-Body Mass Index; TyG-WC, TyG-Waist Circumference; TyG-WHtR, TyG-Waist-to-Height Ratio; UA, Uric Acid; VAI, Visceral Adiposity Index; VFA, Visceral Fat Area; WC, Waist Circumference; WHR, Waist-to-Hip Ratio; ucOC, Undercarboxylated Osteocalcin.

## Discussion

**Figure 2:**
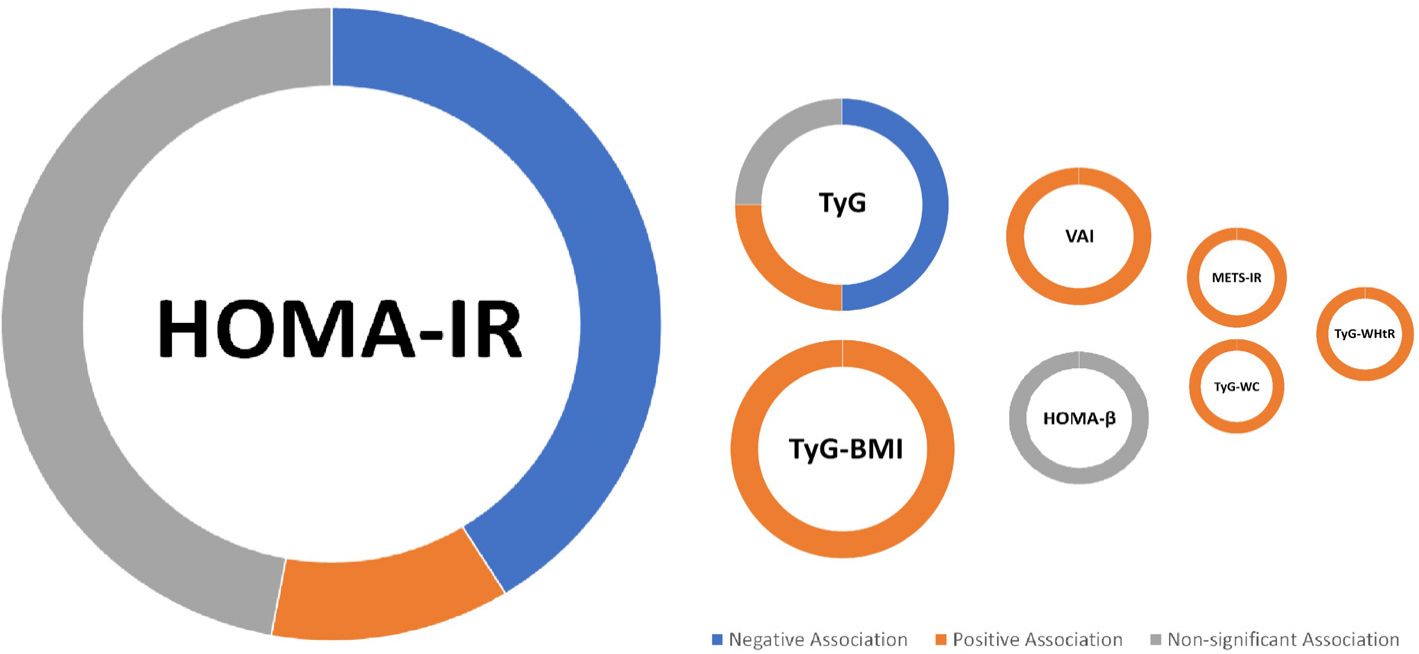
Summary of findings; For the IR index, a larger circle indicates a greater number of included studies (e.g., the largest circle belongs to HOMA-IR, on which the highest number of studies has been conducted (17 studies).

A total of 27 cohorts and cross-sectional studies consisting of 71,525 participants were included in this systematic review to evaluate the possible association of insulin resistance surrogate indices such as HOMA (IR and β), TyG and its derivates (including TyG-BMI, TyG-WtHR, and TyG-WC), as well as METS-IR and VAI, with BMD of different sites. The HOMA index, which has been examined excessively in several studies, appears to have no association with BMD. The majority of the literature indicates that a higher TyG index is linked to a lower BMD. Although other insulin resistance indices (TyG derivates, METS-IR, and VAI) have been investigated in few studies and there is limited evidence of their association, they have a positive association with BMD (**Figure 2**).

Insulin resistance, defined as peripheral tissue’s failure to respond to insulin, is a key feature of metabolic syndrome and an increasing risk factor for BMD loss (48, 49). Conversely, insulin promotes osteoblast proliferation and survival, resulting in increased bone mass. Because insulin resistance and hyperinsulinism are major causes of diabetes, bone mass could be increased in type 2 diabetic patients (50, 51). Therefore, insulin resistance could conceivably have mixed effects on bone mass. In IR, insulin signaling in osteoblasts is impaired, reducing their activity and leading to decreased bone formation (52). Additionally, IR promotes chronic inflammation, elevating pro-inflammatory cytokines like TNF-α and IL-6, which stimulate osteoclasts (bone-resorbing cells), accelerating bone resorption (53). Elevated levels of advanced glycation end products (AGEs) in IR further degrade bone quality by impairing collagen structure, exacerbating bone fragility (54). Insulin resistance promotes the differentiation of mesenchymal stem cells into adipocytes rather than osteoblasts within the bone marrow. This shift increases marrow adipose tissue (MAT), which negatively correlates with BMD and contributes to overall bone loss. The presence of excess adipose tissue in the marrow can disrupt the delicate balance between bone formation and resorption (55). Elevated glucose levels can further impair osteoblastogenesis by activating pathways that promote adipogenesis while inhibiting the expression of key osteogenic transcription factors like Runx2. This dual effect exacerbates the decline in bone mass associated with IR (56). Due to the lack of a standardized definition of insulin resistance, various indices with different components have been established that can probably predict it and are widely used in studies (57).

The HOMA-IR and HOMA-β homeostasis models rely on basal plasma insulin and glucose measurements to assess insulin resistance and pancreatic β cell activity, respectively (58). They have been widely validated and utilized in clinical and epidemiological investigations (59–61). The triglyceride and glucose index, also known as the TyG index, is another insulin resistance surrogate index that has moderate predictive accuracy (62). The TyG index’s accuracy can be improved by combining it with adiposity indicators such as BMI, WC, and waist-to-height ratio, resulting in TyG-BMI, TyG-WT, and TyG-WtHR (63, 64). As for TyG-BMI, high triglycerides mechanistically lead to lipotoxicity by accumulating in non-adipose tissues, which impairs insulin signaling and disrupts pancreatic β-cell function, increasing IR. High glucose levels also heighten oxidative stress by raising reactive oxygen species (ROS) levels, which harm β-cells. Additionally, excess visceral fat—often reflected in elevated TyG-BMI—contributes to chronic low-grade inflammation, further inhibiting insulin pathways. The TyG-WtHR combines TyG with waist-to-height ratio, highlighting central obesity’s role in IR. Central obesity elevates circulating free fatty acids, impairing insulin signaling in muscles and liver. This also leads to inflammation, oxidative stress, and reduced metabolic flexibility—the body’s ability to alternate between fat and carbohydrate oxidation—which disrupts glucose homeostasis. Lastly, the TyG-WC index incorporates waist circumference to assess IR risks tied to abdominal obesity. High waist circumference, linked to greater visceral fat, increases pro-inflammatory cytokine secretion, contributing to systemic IR. It also correlates with altered lipid metabolism, with elevated triglycerides and reduced HDL cholesterol worsening insulin sensitivity.

The visceral adiposity index, or VAI, is another insulin resistance surrogate composed of BMI, WC, triglyceride, and HDL levels, VAI is another metric that probably predicts insulin resistance (65). The METS-IR index incorporates components of metabolic syndrome, including waist circumference, blood pressure, fasting glucose, and lipids, to gauge IR. This syndrome affects IR through adipokine dysregulation, where altered adipokine secretion (e.g., elevated leptin and reduced adiponectin) fosters systemic inflammation and leptin resistance, leading to hyperglycemia. It also induces endothelial dysfunction, reducing nitric oxide and impairing glucose uptake (66). Although the variety of insulin resistance surrogates and their components enables the assessment of many aspects of insulin resistance in individuals, it also brings significant heterogeneity in their implications and associated expected outcomes, such as BMD. The next section attempts to construct a framework for the interpretation and implementation of each insulin resistance surrogate in predicting BMD.

Among studies that evaluated the association between HOMA-IR and BMD, 8 found no association, 7 found a negative association, and 2 found a positive association. A total of two studies that evaluated the association between BMD and HOMA-β also indicated no association. Apparently, the formulas of HOMA-IR and HOMA-β, which contain fasting plasma glucose and insulin, can be valid and reliable in the prediction of insulin resistance (67). However, they failed to estimate BMD accurately, as the majority of studies indicated no association between them.

The review of the studies that assessed the TyG index and BMD association identified three negative associations, two no associations, and one positive association. As a result, the serum triglyceride and glucose index, which has moderate to low accuracy in diagnosing insulin resistance (62), is unlikely to be associated with BMD. However, when the details of the results were reviewed, it turned out that all three studies that found a negative relationship between TyG and BMD investigated whole-body BMD. None of the studies that found no association between TyG and BMD did so with whole-body BMD. This topic could be the focus of future research, as three studies may not be enough to provide evidence of a negative association between TyG and whole-body BMD.

TyG-BMI was found to be positively associated with BMD in three studies, while TyG-WtHR and TyG-WC were positively associated with BMD in one study. The potential positive correlation between TyG-BMI and higher BMD could be explained by incorporating BMI into the TyG formula, as increased body weight increases mechanical load and activates osteoblasts, and each unit increase in BMI is associated with a 0.0082 g/cm2 increase in BMD (68–70). Two studies found a positive connection between VAI and BMD, while one found a favorable association between METS-IR and BMD. Although there is limited evidence of an association between BMD, METS-IR, and VAI, this can be explained by the fact that both indices include an integrated BMI component (the VAI formula contains HDL, WC, triglyceride, and BMI, and the METS-IR formula contains fasting glucose, fasting triglyceride, HDL-c, and BMI).

This study carried several limitations. First, the variety in populations and insulin resistance indices across the included studies precluded the possibility of performing a meta-analysis. Additionally, the cross-sectional nature of most included studies restricts the ability to infer causality between insulin resistance and BMD. Variability in the covariates adjusted for in the different studies may also introduce bias, as some relevant confounders might not have been consistently accounted for. Furthermore, the diverse characteristics of the sample size of studies in sex, physiologic parameters (e.g., menstruation status), and underlying disease contribute to the heterogeneity of studies on the association between insulin resistance surrogates and BMD. Another limitation is that the relationship between insulin resistance indices and specific bone quality parameters, such as bone microarchitecture and strength, has not been explored in the included studies, calling for further research focusing on how insulin resistance affects bone health beyond bone mineral density. To establish a firm conclusion, the link between insulin resistance indices and restricted evidence, particularly the TyG index, should be investigated in cohort studies.

## Conclusion

Despite the heterogenicity of studies on the association between the HOMA index and BMD, there is probably no association. Although there are few studies on other insulin resistance surrogate indices, TyG may have a negative association with whole BMD. Other insulin resistance indices, such as TyG derivates, VAI, and METS-IR, are observed to have positive associations, which may be due to the addition of BMI into their formula. Future research should prioritize conducting longitudinal studies in order to explain causation and gain a deeper understanding of the mechanisms involved in these correlations. Research should investigate how lifestyle interventions, like diet and exercise, can affect insulin resistance levels and their influence on bone health.

## Data Availability

The original contributions presented in the study are included in the article/**Supplementary Material**. Further inquiries can be directed to the corresponding author.
