# Supplementary material for: A systematic review of the association between insulin resistance surrogate indices and bone mineral density
Source: Front Endocrinol (Lausanne). 2024 Dec 18;15:1499479. doi: 10.3389/fendo.2024.1499479 (PMC11688183; doi:10.3389/fendo.2024.1499479)
Supplement: Supplementary file 1 [file Table1.docx]

| **Table S1.** Quality assessment of cross-sectional studies | | | | | | | | |
| --- | --- | --- | --- | --- | --- | --- | --- | --- |
| **Author, year** | **Q1** | **Q2** | **Q3** | **Q4** | **Q5** | **Q6** | **Q7** | **Q8** |
| Kim, 2013 | Yes | Yes | Yes | Yes | Yes | Yes | Yes | Yes |
| Shin, 2014 | Yes | Yes | Yes | Yes | Yes | Yes | Yes | Yes |
| Srikanthan, 2014 | Yes | Yes | Yes | Yes | Yes | Yes | Yes | Yes |
| Shanbhogue, 2016 | Yes | Yes | Yes | Yes | Yes | Yes | Yes | Yes |
| Choo, 2017 | Yes | Yes | Yes | Yes | Yes | Yes | Yes | Yes |
| Seoung, 2018 | Yes | Yes | Yes | Yes | Yes | Yes | Yes | Yes |
| Yang, 2019 | Yes | Yes | Yes | Yes | Yes | Yes | Yes | Yes |
| de Araújo, 2020 | Yes | Yes | Yes | Yes | Yes | Yes | Yes | Yes |
| Wang, 2020 | Yes | Yes | Yes | Yes | Yes | Yes | Yes | Yes |
| Campillo-Sánchez, 2020 | Yes | Yes | Yes | Yes | Yes | Yes | Yes | Yes |
| Yoon, 2021 | Yes | Yes | Yes | Yes | Yes | Yes | Yes | Yes |
| Giudici, 2021 | Yes | Yes | Yes | Yes | Yes | Yes | Yes | Yes |
| Ye, 2023 | Yes | Yes | Yes | Yes | Yes | Yes | Yes | Yes |
| Sun, 2023 | Yes | Yes | Yes | Yes | Yes | Yes | Yes | Yes |
| Pu, 2023 | Yes | Yes | Yes | Yes | Yes | Yes | Yes | Yes |
| Chen, 2023 | Yes | Yes | Yes | Yes | Yes | Yes | Yes | Yes |
| Zhan, 2023 | Yes | Yes | Yes | Yes | Yes | Yes | Yes | Yes |
| Xuan, 2024 | Yes | Yes | Yes | Yes | Yes | Yes | Yes | Yes |
| Tian N, 2024 | Yes | Yes | Yes | Yes | Yes | Yes | Yes | Yes |
| Chen, 2024 | Yes | Yes | Yes | Yes | Yes | Yes | Yes | Yes |
| Tian C, 2024 | Yes | Yes | Yes | Yes | Yes | Yes | Yes | Yes |
| Shao, 2024 | Yes | Yes | Yes | Yes | Yes | Yes | Yes | Yes |
| Sretenović, 2021 | Yes | Yes | Yes | Yes | Yes | Yes | Yes | Yes |
| Q1. Were the criteria for inclusion in the sample clearly defined?  Q2. Were the study subjects and the setting described in detail?  Q3. Was the exposure measured in a valid and reliable way?  Q4. Were objective, standard criteria used for measurement of the condition?  Q5. Were confounding factors identified?  Q6. Were strategies to deal with confounding factors stated?  Q7. Were the outcomes measured in a valid and reliable way?  Q8. Was appropriate statistical analysis used? | | | | | | | | |

| **Table S2.** Quality assessment of cohort studies | | | | | | | | | | | |
| --- | --- | --- | --- | --- | --- | --- | --- | --- | --- | --- | --- |
| **Author, year** | **Q1** | **Q2** | **Q3** | **Q4** | **Q5** | **Q6** | **Q7** | **Q8** | **Q9** | **Q10** | **Q11** |
| Iki, 2012 | Yes | Yes | Yes | Yes | Yes | Yes | Yes | Yes | Yes | No | Yes |
| Kalimeri, 2018 | Yes | Yes | Yes | Yes | Yes | Yes | Yes | Yes | Yes | No | Yes |
| Napoli, 2019 | Yes | Yes | Yes | Yes | Yes | Yes | Yes | Yes | Yes | Yes | Yes |
| Wen, 2022 | Yes | Yes | Yes | Yes | Yes | Yes | Yes | Yes | Yes | Yes | Yes |
| Q1. Were the two groups similar and recruited from the same population?  Q2. Were the exposures measured similarly to assign people to both exposed and unexposed groups?  Q3. Was the exposure measured in a valid and reliable way?  Q4. Were confounding factors identified?  Q5. Were strategies to deal with confounding factors stated?  Q6. Were the groups/participants free of the outcome at the start of the study (or at the moment of exposure)?  Q7. Were the outcomes measured in a valid and reliable way?  Q8. Was the follow up time reported and sufficient to be long enough for outcomes to occur?  Q9. Was follow up complete, and if not, were the reasons to loss to follow up described and explored?  Q10. Were strategies to address incomplete follow up utilized?  Q11. Was appropriate statistical analysis used? | | | | | | | | | | | |
